# Post Reduction Mammaplasty Pyoderma Gangrenosum: An Unusual Presentation of a Misdiagnosed Entity

**DOI:** 10.7759/cureus.11432

**Published:** 2020-11-11

**Authors:** Jesus Zapata Alvarez, Alfredo Patrón Gómez

**Affiliations:** 1 Internal Medicine, Universidad de Antioquia, Medellin, COL; 2 Plastic Surgery, Universidad de Antioquia, Medellin, COL

**Keywords:** pyoderma gangrenosum, reduction mammaplasty, breast surgery, surgical complications

## Abstract

Pyoderma gangrenosum is a rare skin disorder that could be triggered after surgical trauma. However, more uncommon, this condition could follow breast surgery. An opportune diagnosis and adequate treatment are essential in order to avoid wound chronicity and aesthetic sequels. We report a 51-year-old woman who presented multiple wound complications after a reduction mammaplasty. This particular case is unique, because bilateral and unusual presentation after another uncomplicated previous surgical procedure, and illustrates how a patient could be taken through different rational surgical therapeutic modalities without improvement, and deterioration of clinical picture. Finally, a diagnosis of Pyoderma gangrenosum was established, and immunosuppressive treatment was completed, with an appropriate resolution of this problematic condition.

## Introduction

Pyoderma gangrenosum (PG) is a rare inflammatory skin disorder of unknown etiology mainly characterized by cutaneous papillomatous pustules that evolve rapidly into large, painful, necrotic ulcers with unclear edges [1,2]. PG has an estimated incidence of three to ten per million per year [3], and it was initially described by Brock in 1916 and subsequently by Brunsting et al. in 1930. Since then, PG has been recognized to have five clinical presentations, including ulcerative PG, bullous PG, pustular PG, vegetative PG and peristomal PG [4].

In 2007, Ouazzani et al. [1] introduced a new clinical entity known as post-surgical PG (PSPG), that refers to a compromise of surgical incisions within the immediate postoperative period [2]. This condition has been more frequently reported in adults (95-96% of cases), and predominantly in females. Main affected body areas are breasts (25%), chest wall (14%) and abdomen (14%) [5]. Several authors have reported PSPG cases in breast surgery, most of them after reduction mammoplasties [2,6]. This case is very particular because our patient had already undergone a surgical procedure without triggering PSPG; also, there were employed management strategies not described previously for this condition.

This case report aims to emphasize in strict clinical supervision of wound-healing complications after breast reduction surgery, in order to establish an accurate distinction among PSPG, wound dehiscence and surgical site infection, because of their different algorithms of treatments and devastating clinical scenarios with inaccurate treatments.

## Case presentation

**Figure 1:**
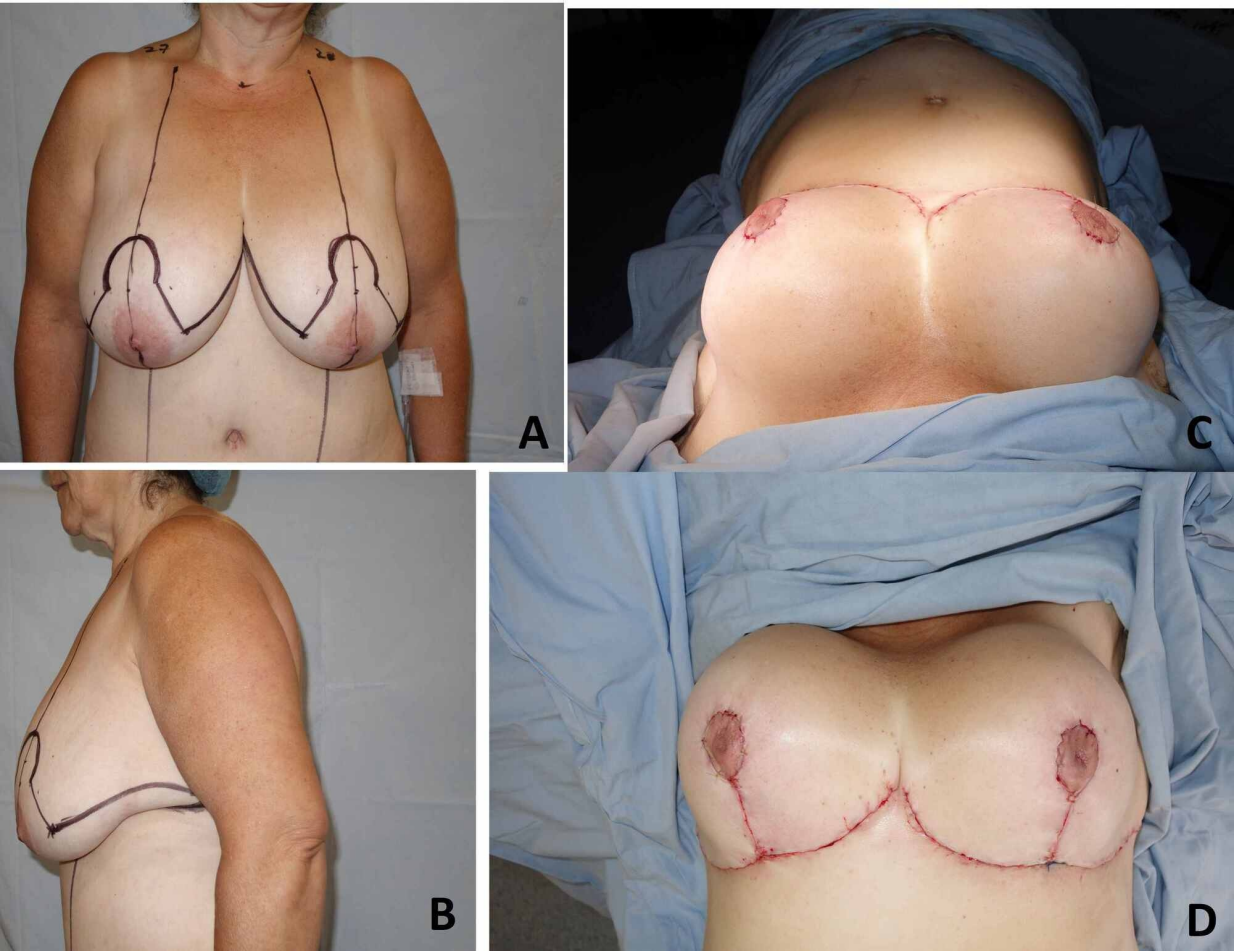
Pre and Postoperative pictures of uncomplicated reduction mammoplasty. (A-B) Preoperative planning. (C-D) Immediate Postoperative.

A 51 years old obese woman had undergone a gastric bypass in 2008. An uncomplicated post-bariatric abdominoplasty was performed in March 2014 because of the excessive abdominal apron. Three months later, the patient consulted for breast surgery, complaining of dorsalgia and back pain attributed to breast weight. A physical exam, breast enlargement and asymmetry were documented, with a right sternal notch-nipple distance of 28cm, and left sternal notch-nipple distance of 27cm, grade III bilateral breast ptosis, and breast hypertrophy. A standard, inverted T scar, superior pedicle reduction mammaplasty was performed without intraoperative complications (Figure 1).

**Figure 2:**
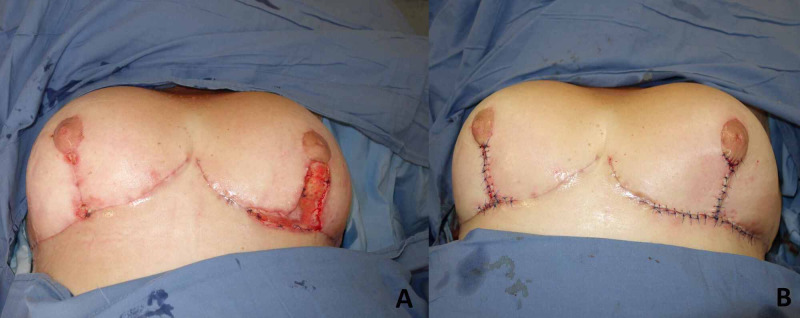
Pre and Postoperative first dehiscence repair of surgical breast wounds. (A) Dehiscences. (B) Repair of surgical breast wounds.

On day 20 after surgery, major dehiscence was documented in both vertical and horizontal wounds of left mammaplasty, and the join area of vertical with a horizontal wound in the right side. Nipple areola complexes (NAC) remained without vascular compromise. Subsequently, the patient was reoperated, with wound debridement and re-suture of mammaplasty flaps, achieving good skin closure. The patient was discharged with a routine post-op appointment. (Figure 2).

**Figure 3:**
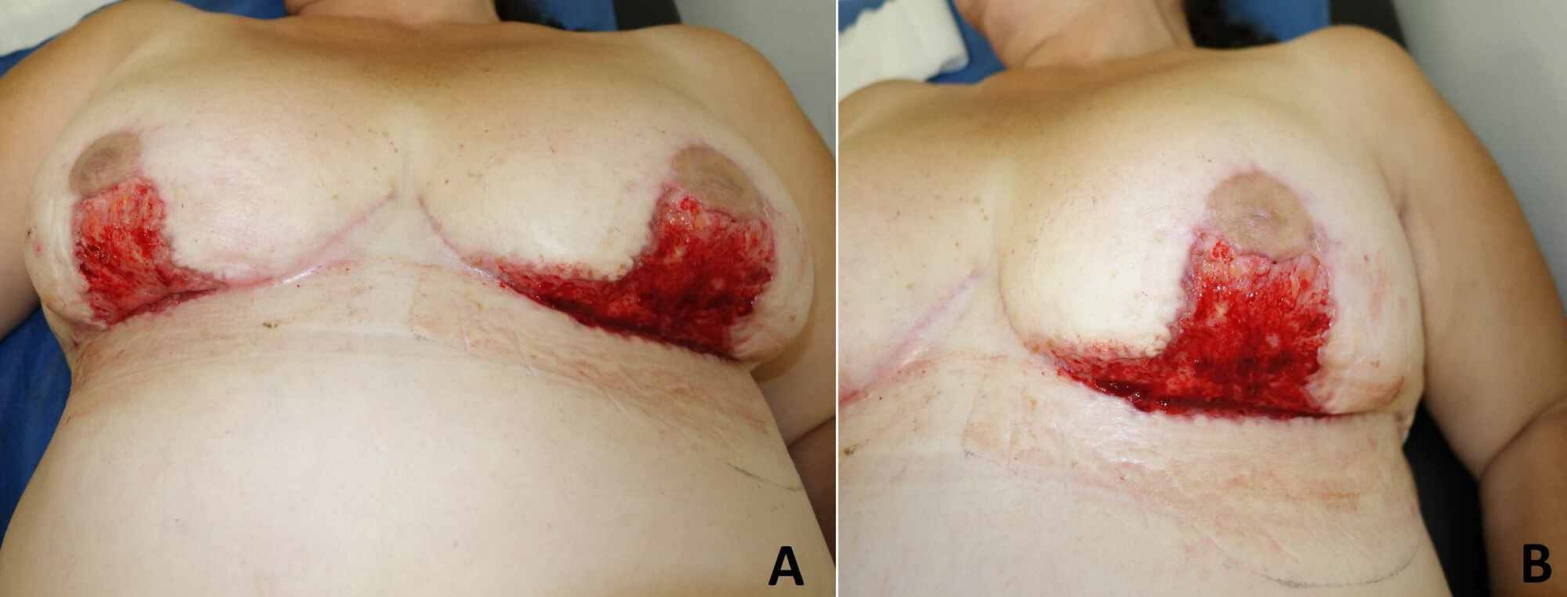
Second dehiscence. Left breast is shown closer. Note sparing of Nipple areola complexes (NAC) (A) Breasts dehiscences. (B) Left Breast dehiscence.

**Figure 4:**
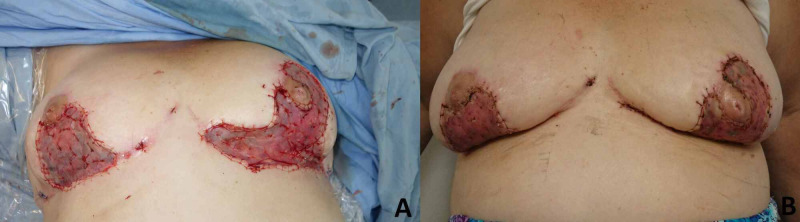
Intra and Postoperative view of partial-thickness skin grafts. (A) Intraoperative view. (B) Postoperative view. Source: Author

Nevertheless, after one month, she returned to medical consultation because of wound dehiscence again in all skin sutures from both breasts, and extension to previous uncompromised areas, predominantly in the left side. (Figure 3). Wound cultures were made with negative results. The patient’s nutritional state was also evaluated. Low levels of prealbumin, transferrin and total blood proteins were found. Intra-hospital management was made to guarantee adequate supplementation. For breasts wound closure, split-thickness skin grafts were performed, to avoid suture skin tension and new dehiscence (Figure 4). After the second reoperation, oral vitamins supplementation was prescribed, and ambulatory consultation one week later assessed adequate evolution of skin grafts.

**Figure 5:**
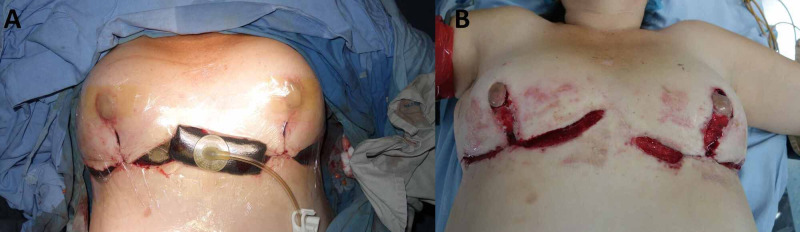
Vacuum-Assisted Closure (VAC) therapy applied after partial-thickness skin grafts loss. (A) VAC therapy. (B) Wounds improvement after VAC therapy.

Patient came for the third time one month later, with mammaplasty wounds dehiscence in both breasts, showing more severe compromise in the left side, also with serous secretion, erythema and poor cicatrization in skin graft donor thigh area. Due to the extremely complicated management of these wounds, inpatient Vacuum-Assisted Closure (VAC) therapy was used in both breast wounds, and Dermatology consultation was requested (Figure 5).

**Figure 6:**
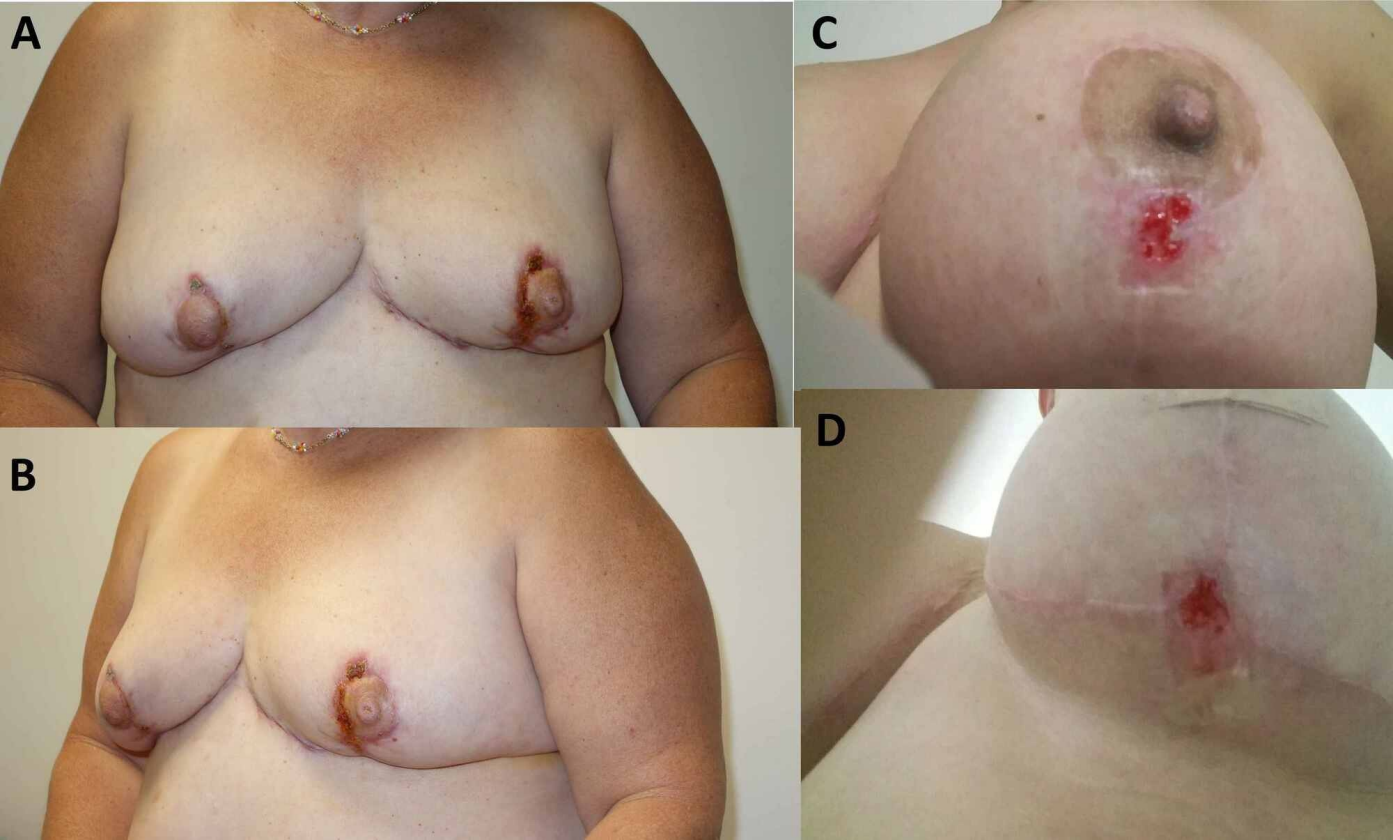
Long term skin healing after systemic and local corticosteroid therapy. (A-D) Skin healing and evolution.

Dermatology considered Pyoderma gangrenosum (PG) as a potential diagnosis and decided to initiate treatment with oral corticosteroids. A skin biopsy from an injury site was done, with nonspecific inflammatory results. Following several weeks, patient wounds improved significantly, erythema and secretion were completely resolved, showing almost complete healing in breast wounds and thigh donor sites, without further surgical intervention (Figure 6). Afterwards, the patient continued with satisfactory wound healing and scar evolution.

## Discussion

Surgery triggered PSPG is extremely uncommon. Clinical signs are usually revealed between four days to six weeks in the post-op period, and females are more frequently affected. PSPG has been reported predominantly in the breast but also related to cardiothoracic, abdominal, orthopaedic, obstetric and gynaecological surgery [2]. Considering different types of breast surgery, most cases have been reported after reduction mammoplasty, but despite this, there are still less than 50 cases of post-reduction mammoplasty PG reported in the world. Generally, the entity was triggered after the first surgery, in contrast with our case presentation, who had previous surgical interventions before developing the disease. A systematic review by Ehrl et al. included a total of 68 articles, reporting 87 cases of PSPG following aesthetic and reconstructive breast surgery, 44% of them occurred after a breast reduction [1]. Kevin et al. reported 220 cases of PSPG among all body areas, identifying 56 cases in the breast region, including 25 cases after reduction mammaplasty [6].

Pathophysiology of this condition remains poorly understood; it is theorized as an intricate autoimmune reaction pattern with either multiple pathways that creates a heterogeneous disease presentation and course. As described in this case, the initial presentation of PSPG is characterized by erythema of the surgical site, pain and eventually wounds dehiscence, with progressive worsening. Furthermore, areas of skin ulceration around necrotic centres with undermined violaceous edges could be present [1,7]. PSPG has been associated with autoimmune disorders, including rheumatoid arthritis (RA), lupus, pemphigus and hypothyroidism, although 50% of cases do not have associated disease identified at the time of presentation. Because PSPG is located in surgical wounds early in the postoperative stage, it is commonly diagnosed as wound infection and treated ineffectively with antibacterial agents and debridement without clinical improvement [6], allowing progression with further skin compromise [5].

Diagnosis of PSPG is made by exclusion after the failure of antibiotic therapies and surgical debridement [8], based on clinical presentation. To date, there are no gold standard laboratory tests. Usually, Skin biopsies findings are nonspecific. As a result, in 2004, Sue et al. proposed some diagnostic criteria that could guide clinicians to rule out this disease. However, our patient only fulfilled only one of these criteria (adequate response to corticosteroid treatment) [2].

The choice of adequate treatment varies according to several features, including number and size of lesions, compromised area, extracutaneous involvement, presence of associated diseases, and side effects of treatment. Optimal management encompasses avoidance of triggers, appropriate wound care, adequate pain management in addition to systemic, topical or targeted immunomodulatory therapies [4]. Prednisone has been proposed as first-line therapy, and cyclosporine or dapsone could be used as alternatives. Also, topical tacrolimus has shown some benefit in the treatment of PSPG [5]. In our case, as previously described, treatment with oral corticosteroids plus intradermal triamcinolone injections, showed satisfactory response despite severe skin compromise [9]. Usually, disease improvement is dramatic, with immediate resolution of erythema, edema and pain relief; in addition, once immunomodulatory therapy is given, split-thickness skin grafts can be performed to replace coverage [5,7]. In our view, this case was worsened by a deficient nutritional condition related to previous gastric bypass, and unsuspected PG as a primary diagnosis, because of previous uncomplicated abdominoplasty that healed uneventfully. Also, we consider that VAC assisted closure had a significant influence in achieving an acceptable aesthetic result. This report demonstrates that PG could affect multiple random areas in the same patient, despite several surgical sites, without proven systemic immunological diseases. PG should be ruled out in postoperative problematic wound healing complications of breast surgery, before utilizing complex surgical treatment modalities.

## Conclusions

Post reduction mammaplasty PG is certainly an uncommon, misdiagnosed entity, with less than 50 cases reported in the world literature. This condition would be considered as a differential diagnosis in patients with multiple dehiscences or recalcitrant wound complications after breast lift or reduction surgery, regardless of the personal history of autoimmune diseases. Early suspicion allows adequate management, preventing prolongation of skin compromise and functional or aesthetic sequels. More studies are needed to clarify the role of VAC assisted closure and intradermal corticosteroids in addition to standard therapy.
